# Highlights of the 2021 American Joint Replacement Registry Annual Report

**DOI:** 10.1016/j.artd.2022.01.020

**Published:** 2022-01-29

**Authors:** Ahmed Siddiqi, Brett R. Levine, Bryan D. Springer

**Affiliations:** a Orthopaedic Institute Brielle Orthopaedics, Manasquan, NJ, USA; b Assistant Professor, Hackensack Meridian Health, Hackensack, NJ, USA; c Associate Professor, Rush University Medical Center, Chicago, IL, USA; d OrthoCarolina Hip and Knee Center, Department of Orthopedics Atrium Musculoskeletal Institute, Charlotte, NC, USA

## American Joint Replacement Registry 2021 executive summary

In 2017, the American Joint Replacement Registry (AJRR) became the first registry to join the American Academy of Orthopaedic Surgeons (AAOS) Registry Program. AJRR continues to work toward the AAOS Registry objectives under the supervision of the AAOS Registry Oversight Committee and the AJRR Steering Committee. Since then, the AAOS Registry Program has grown to include registries from a variety of anatomic sites and orthopedic specialty areas, including the Shoulder & Elbow Registry, the Musculoskeletal Tumor Registry, the American Spine Registry—a collaborative registry with the American Association of Neurological Surgeons—and the recently announced 2021 Fracture & Trauma Registry. For AJRR, 2020-2021 has been defined by a slew of accomplishments and expansion. This annual report comprises almost 2.4 million hip and knee surgeries from over 1150 institutions that submitted data, representing an 18.3% increase in total procedural volume compared with that in 2020. This represents a 40% capture rate of all United States total joint arthroplasty (TJA) volume annually. A great deal of effort has been put into ensuring that the AJRR retains its position as the definitive national registry for US joint arthroplasty surgeons.

Historically, ambulatory surgery centers (ASCs), both freestanding and affiliated with hospitals, have not had a prominent presence in AJRR reports, as most of the procedural information in the registry had originated from hospital-based procedures. The AAOS started developing initiatives to better assist ASCs and private practices to allow easier accessibility to data quality, analysis, and benchmarking. AAOS has launched a partnership with the Ambulatory Surgery Center Association to implement a pilot program that offers data submission guidelines required for ASCs with low volume or limited technological capabilities. As a result, the number of reports on procedures submitted by ASCs climbed exponentially between 2012 (n = 5) and 2020 (n = 14,281), and, remarkably, the number of procedures submitted by ASCs has risen by 82% since the publication of the 2020 AJRR Annual Report. Patient-reported outcome measures (PROMs) have garnered increased attention over recent years as they must be reported at various levels for many current alternative payment models. AJRR collects PROMs and encourages sites to submit these data at set intervals: a baseline measure obtained before the surgery, a measure at 90 days postoperatively, and a subsequent measure at 1 year postoperatively. AJRR provides a PROM platform inside RegistryInsights at no extra cost to assist institutions with PROM data collection. This platform provides PROM capture and storage services and is available to all AJRR institutions both preoperatively and postoperatively. Alternately, participating institutions can also continue to use their current PROM programs if they so choose. As of 2019, AJRR recommends and supports on their PROM platform the collection of Hip dysfunction and Osteoarthritis Outcome Score for Joint Replacement, Knee injury and Osteoarthritis Outcome Score for Joint Replacement, Patient-Reported Outcomes Measurement Information System-Global 10, and Veterans RAND 12-Item Health Survey. Other PROMs are collected but not used for analyses. As of December 31, 2020, 290 sites out of 1152 (25.2%) have submitted PROMs, which is a 39% increase in sites compared with the previous 2020 AJRR Annual Report. Based on 8241 matched Hip dysfunction and Osteoarthritis Outcome Score for Joint Replacement scores, 93% of patients achieved a meaningful improvement after elective primary total hip arthroplasty (THA). Similarly, based on the 14,127 matched Knee injury and Osteoarthritis Outcome Score for Joint Replacement scores, 88% of patients achieved a meaningful improvement after total knee arthroplasty (TKA). As the case volume grows, these matched data points will be increasingly important to set benchmarks for hip and knee replacement outcome reporting.

Tracking and longitudinal monitoring of outcomes remains an emphasis of the AAOS Registry program. RegistryInsights has been further expanded and improved to assist with participating sites' access to their own real-time dashboards while allowing direct comparison to AJRR national benchmarks. Finally, for those who want more customized capabilities, AJRR provides institutional or surgeon-specific custom reports upon request.

Over the last year, AJRR has placed an ongoing strong emphasis on publications and presentations based on AJRR data. *Journal of Arthroplasty*, *Journal of the American Academy of Orthopedic Surgeons*, and *Clinical Orthopedics and Related Research* are just a few of the peer-reviewed publications where AJRR has had articles published. The American Association of Hip and Knee Surgeons, International Society of Arthroplasty Registries, The Knee Society, The Hip Society, North Carolina Orthopedic Association, and the Musculoskeletal Infection Society have all had podium presentations and posters based on AJRR data at their respective annual meetings in 2020 and 2021. Patient-reported outcome metrics, infection, arthroplasty for femoral neck fractures, patient migration, and dual mobility are among the most published topics.

## 2021 AJRR Annual Report highlights

The 2021 AJRR Annual Report reported on 2,244,587 primary and revision hip and knee arthroplasties between 2012 and 2020. The majority of the surgeries were primary TKA (54.5%) and primary THA (38.6%). Female patients represented 58.5% of all procedures while male patients represented 38.6% of cases. THA patients were on average 66.1 years old, whereas TKA patients were on average 67 years old. Although race was not recorded in 15.8% of instances, most patients were Caucasian (75.6%). The mean 2020 procedure count for AJRR surgeons conducting elective primary THA and TKA was 26.7 and 33.9, respectively. Interestingly, the case per surgeon median is within the lower quartiles, suggesting a higher frequency of lower volume surgeons reporting to the registry. This procedure distribution matches prior studies of TJA in the United States [1,2].

Many trends identified in previous AJRR Annual Reports were maintained in the 2021 report. Ceramic head usage is increasingly common in elective THA, while there has been a corresponding and statistically significant decrease in cobalt-chromium usage (*P* < .0001). The increase in ceramic head use is likely explained by concerns over trunnion and taper corrosion seen with cobalt-chromium heads. Less than 16% of elective primary THA were performed using a metal-on-polyethylene articulation in 2020, while ceramic-on-polyethylene remains the dominant bearing couple choice. In both primary and revision THAs, the use of dual-mobility constructs has increased. While hemiarthroplasties remain the most common procedure for femoral neck fractures, THA is becoming increasingly more popular. For both elective primary THA and THA for femoral neck fractures, the use of cement for femoral component fixation is gradually increasing. However, the use of cemented femoral component fixation in the AJRR (4.2%) remains lower than that seen in international registries (National Joint Registry 32.3%, Australian Orthopedic Association National Joint Replacement Registry 39.2%, Swedish Hip Arthroplasty Registry 58%) [[3], [4], [5]].

For primary TKA, more than half of all procedures used posterior-stabilized components until 2019 when the rate dropped below 50%. The use of cruciate-retaining designs has increased annually since 2016 to reach 46.2% in 2020. The use of ultracongruent components has similarly increased between 2012 and 2019. This trend can likely be attributed to ultracongruent and cruciate-retaining designs demonstrating significantly reduced cumulative percent revision compared with posterior stabilized designs after adjusting for age and sex in patients aged ≥65 years as reported to either AJRR or Centers for Medicare and Medicaid Services (CMS).

Although cemented TKA fixation remains predominant, the use of cementless fixation in primary TKA is rapidly increasing in the AJRR and was reported for over 14% of all primary knee arthroplasties in 2020. This increasing trend is also similarly reported in the Swedish Knee Arthroplasty Registry (8%) and National Joint registry (4.2%) [3,6]. Cementless fixation was found to have a significant decrease in cumulative percent revision compared with cemented fixation in male patients aged ≥65 years in AJRR and CMS databases (*P* = .0023) and in patients younger than 65 years reported to AJRR (*P* = .0044). Medial or lateral unicompartmental knee arthroplasty use has decreased in prevalence since 2012 and represented 2.7% of all primary knee arthroplasties reported to AJRR in 2017. Most recently, there has been a slight bump in unicompartmental knee arthroplasty procedural volume with an increase to 4.2% in 2020.

For both THA and TKA, postoperative length of stay in the AJRR cohort has continued to decrease with a substantial decrease in nonhome discharge (representing <6% of all discharges). The utilization of general anesthesia has declined for both THA and TKA, with an increase in regional and neuraxial anesthesia. The relationship between patient characteristics such as demographics and comorbidities and the risk of revision after initial TKA and THA has been a focus of AJRR’s research. The 2021 Annual Report used age by decade for those aged 65 years and older to further investigate risk of revisions based on age. A trend was identified suggesting older age was associated with increased cumulative percent revision. This trend was statistically significant comparing male patients older than 84 years to those aged 65-74 years (*P* < .0001) and comparing female patients aged 75-84 years to those aged 65-74 years (*P* = .0105). In contrast to the THA analyses, the younger age group (65-74 years) was found have a significantly higher cumulative percent of revision than the older age groups for elderly TKA patients. However, additional variables that may impact the probability of failure and revision surgery are not taken into consideration in these analyses.

Finally, the purpose of each annual report is to improve analytics and understand trends. Analyses based on registry data are expected to improve as AJRR grows. This year, cumulative percent revision curves with a diagnostic specific endpoint were created for the first time, analyzing revision due to infection for TKA and revision owing to periprosthetic fracture for THA patients older than 65 years. This is the fourth year of presenting revision curves over time and implementing additional CMS data. The report also includes device-specific cumulative percent revision estimates stratified by bearing and fixation type in the supplement material. A significant amount of work was carried out on a consensus-driven technique to offer a solid platform for future research, allowing for the development of more complex and thorough survivorship analyses. The data collected in AJRR are a growing avenue for TJA research to further improve procedural value and foster innovation [7].

## Conflicts of interest

The authors declare the following financial interests/personal relationships which may be considered as potential competing interests: A. Siddiqi is a consultant for Zimmer-Biomet and Intellijoint and has stock options in ROMTech. B. R. Levine is a consultant for Exactech and Link; receives royalties from Link, Human Kinetics, SLACK Inc., and Wolters Kluwer; is the Deputy Editor of *Arthroplasty Today*—“have recused myself of the peer review process for this article”. B. D. Springer is a consultant for Stryker and ConvaTec; receives royalties from OsteoRemedies and Stryker; and is a member of the AJRR Steering Committee.

## Appendix A Supplementary data

Conflict of Interest Statement for Siddiqi

Conflict of Interest Statement for Levine

Conflict of Interest Statement for Springer
