# Supplementary material for: Highlights of the 2021 American Joint Replacement Registry Annual Report
Source: Arthroplast Today. 2022 Jan 29;13:205–7. doi: 10.1016/j.artd.2022.01.020 (PMC8810304; doi:10.1016/j.artd.2022.01.020)
Supplement: Conflict of Interest Statement for Siddiqi [file mmc1.pdf]

CONFLICT OF INTEREST STATEMENT

American Association of Hip and Knee Surgeons

(Adopted from the American Academy of Orthopaedic Surgeons disclosure statement)

The following form **must be filled out completely and submitted by each author (example, 6 authors, 6 forms). All items require a response. If there is no relevant disclosure for a given item, enter "*None*."**

Manuscript Title AJRR Update 2021

1. Royalties from a company or supplier (The following conflicts were disclosed)

   None

2. Speakers bureau/paid presentations for a company or supplier (The following conflicts were disclosed)

   None

3A. Paid employee for a company or supplier (The following conflicts were disclosed)

   None

3B. Paid consultant for a company or supplier (The following conflicts were disclosed)

   Zimmer Biomet, Intellijoint

3C. Unpaid consultants for a company or supplier (The following conflicts were disclosed)

   AZ Solutions, LLC

4. Stock or stock options in a company or supplier (The following conflicts were disclosed)

   ROMTech

5. Research support from a company or supplier as a Principal Investigator (The following conflicts were disclosed)

   none

6. Other financial or material support from a company or supplier (The following conflicts were disclosed)

   none

7. Royalties, financial or material support from publishers (The following conflicts were disclosed)

   none

8. Medical/Orthopaedic publications editorial/governing board (The following conflicts were disclosed)

   none

9. Board member/committee appointments for a society (The following conflicts were disclosed)

   none

Each author must sign AND print or type his/her name, date and submit a separate form

In addition, one BLINDED Conflict of Interest form (no author names used) should be submitted per manuscript with all author disclosures.

Ahmed Siddiqi		1/12/2022
Author Name (Print or Type)	Author Signature	Date
