# Supplementary material for: Highlights of the 2021 American Joint Replacement Registry Annual Report
Source: Arthroplast Today. 2022 Jan 29;13:205–7. doi: 10.1016/j.artd.2022.01.020 (PMC8810304; doi:10.1016/j.artd.2022.01.020)
Supplement: Conflict of Interest Statement for Levine [file mmc2.pdf]

INDIVIDUAL CONFLICT OF INTEREST STATEMENT

American Association of Hip and Knee Surgeons

(Adopted from the American Academy of Orthopaedic Surgeons disclosure statement)

The following form **must be filled out completely and submitted by each author (example, 6 authors, 6 forms). All items require a response. If there is no relevant disclosure for a given item, enter "*None.*"**

Manuscript Title: AJRR Update

1. Royalties from a company or supplier (The following conflicts were disclosed)

None

2. Speakers bureau/paid presentations for a company or supplier (The following conflicts were disclosed)

None

3A. Paid employee for a company or supplier (The following conflicts were disclosed)

None

3B. Paid consultant for a company or supplier (The following conflicts were disclosed)

Link, Exactech

3C. Unpaid consultants for a company or supplier (The following conflicts were disclosed)

None

4. Stock or stock options in a company or supplier (The following conflicts were disclosed)

None

5. Research support from a company or supplier as a Principal Investigator (The following conflicts were disclosed)

Institutional support from Zimmer-Biomet

6. Other financial or material support from a company or supplier (The following conflicts were disclosed)

None

7. Royalties, financial or material support from publishers (The following conflicts were disclosed)

Human Kinetics. Kluwers- Wolters and Slack Inc.

8. Medical/Orthopaedic publications editorial/governing board (The following conflicts were disclosed)

JOA, AT (Deputy editor), Orthopedics

9. Board member/committee appointments for a society (The following conflicts were disclosed)

AAHKS—Patient education and research committees; AAOS—ALI 3; MAOA—Education committee

Each author must sign AND print or type his/her name, date and submit a separate form

In addition, one BLINDED Conflict of Interest form (no author names used) should be submitted per manuscript with all author disclosures.

Brett Levine 1/12/2022

Author Name (Print or Type)	Author Signature	Date
